# The Rise and Fall of TRP-N, an Ancient Family of Mechanogated Ion Channels, in Metazoa

**DOI:** 10.1093/gbe/evv091

**Published:** 2015-06-22

**Authors:** Andreas Schüler, Gregor Schmitz, Abigail Reft, Suat Özbek, Ulrich Thurm, Erich Bornberg-Bauer

**Affiliations:** ^1^Institute for Evolution and Biodiversity, University of Muenster, Germany; ^2^Centre for Organismal Studies, University of Heidelberg, Germany; ^3^HEIKA—Heidelberg Karlsruhe Research Partnership, Heidelberg University, Karlsruhe Institute of Technology (KIT), Heidelberg and Karlsruhe, Germany; ^4^Institute for Neurobiology and Behavioural Biology, University of Muenster, Germany

**Keywords:** protein evolution, domain rearrangements, mechanosensation, neurobiology, Cnidaria, nematocyst evolution

## Abstract

Mechanoreception, the sensing of mechanical forces, is an ancient means of orientation and communication and tightly linked to the evolution of motile animals. In flies, the transient-receptor-potential N protein (TRP-N) was found to be a cilia-associated mechanoreceptor. TRP-N belongs to a large and diverse family of ion channels. Its unusually long N-terminal repeat of 28 ankyrin domains presumably acts as the gating spring by which mechanical energy induces channel gating. We analyzed the evolutionary origins and possible diversification of TRP-N. Using a custom-made set of highly discriminative sequence profiles we scanned a representative set of metazoan genomes and subsequently corrected several gene models. We find that, contrary to other ion channel families, TRP-N is remarkably conserved in its domain arrangements and copy number (1) in all Bilateria except for amniotes, even in the wake of several whole-genome duplications. TRP-N is absent in Porifera but present in Ctenophora and Placozoa. Exceptional multiplications of TRP-N occurred in Cnidaria, independently along the Hydra and the Nematostella lineage. Molecular signals of subfunctionalization can be attributed to different mechanisms of activation of the gating spring. In Hydra this is further supported by in situ hybridization and immune staining, suggesting that at least three paralogs adapted to nematocyte discharge, which is key for predation and defense. We propose that these new candidate proteins help explain the sensory complexity of Cnidaria which has been previously observed but so far has lacked a molecular underpinning. Also, the ancient appearance of TRP-N supports a common origin of important components of the nervous systems in Ctenophores, Cnidaria, and Bilateria.

## Introduction

Mechanoreception is the ability to sense any kind of mechanical force such as touch, weight, vibration, or sound. The evolutionary development of sensing and processing of stimuli is key for the emergence of complex traits, such as self-organization of organisms in general and of their behavior. To current knowledge, mechanoreception is accomplished by a small number of alternative mechanisms which are based on highly specialized molecular arrangements (Sachs 1986; Gillespie and Walker 2001; Delmas and Coste 2013). All known mechanisms of neural mechanoreception involve the opening (gating) of a mechanosensitive ion channel, which is a membrane protein with transmembrane helices. Stressing the gating spring by some mechanical force, such as pushing or pulling, activates the open state of the channel. This opening allows an inward flux of cations through the transmembrane domain along their electrochemical gradient, producing a depolarizing electric signal, the receptor potential, which induces nerve impulses or synaptic transmission. There is no indication of a second messenger for chemical signal transmission during mechanoreception. Mechanoreception in Ecdysozoa, which have a cuticle, follows a “push” mechanism which is characterized by compression of the gating spring (Thurm et al. 1983; Liang et al. 2013). Contrary to that, in all other organisms which are not coated by a cuticle, ciliary mechanotransduction follows a negative sensing force and is triggered by a somewhat inverse “pull” mechanism in which the gating spring is deduced to be stressed (Thurm et al. 1998). Apparently, the shift in function from the more ancestral state of pull to push occurred with the evolution of a cuticle to be shed, at the split of *Ecdysozoa* and *Lophotrochozoa* (see also supplementary fig. S1, Supplementary Material online, for schemata).

The metazoan TRP (transient receptor potential) channel family of proteins comprises seven subfamilies. Some of these subfamilies are involved in mechanoreception, whereas others sense temperature, taste, smell, and pain (Pedersen et al. 2005; Venkatachalam and Montell 2007). TRP-A (for ankyrin), for example, functions as stress sensor and possibly as thermal sensor and was reported to occur in several copies in most metazoans (Venkatachalam and Montell 2007). TRP proteins are, like many other proteins, modularly composed of multiple domains (Moore et al. 2008). All TRP proteins contain a six-transmembrane domain. Several subfamilies, for example, TRP-A and TRP-N, contain repeats of ankyrin domains at their respective N-termini (see Materials and Methods for an overview with functional description of subfamilies).

TRP-N was first described as nompC (for no mechanoreceptor potential C) in the fly *Drosophila melanogaster* (Walker et al. 2000). There, TRP-N is located in the mechanosensory cells of bristle, hair and campaniform sensilla and Johnston’s hearing organ (Eberl and Boekhoff-Falk 2007; Liang et al. 2011). In these cells, TRP-N is bound to the microtubular skeleton of modified cilia in their stimulus-receiving tip.

**Fig. 1.—:**
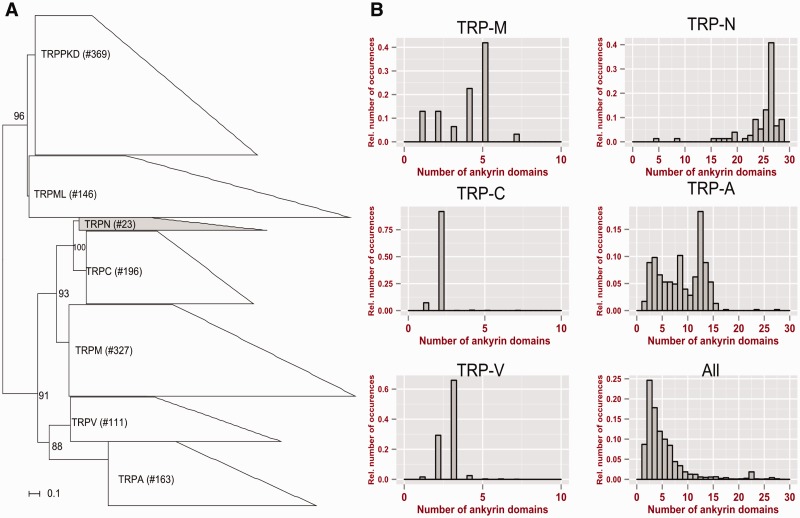
Phylogeny of TRP protein families (*A*) and distributions of ankyrin domains in TRP proteins (*B*): Numbers at nodes indicate bootstrap supports and size of the polygons scales to the size of the families across all used genomes (see Materials and Methods for details). The bar plots in (*B*) show the distribution of ankyrin domains in proteins of the respective TRP families. “All” refers to all proteins, including all TRP proteins, from GenBank with at least one ankyrin domain. The bar plots are based on data from GenBank only and do not include our manually corrected gene models.

TRP-N has a long N-terminal ankyrin-repeat which comprises approximately 28 ankyrin domains. This repeat has been conjectured to be the gating spring which is involved in the transmission of mechanical force to the transmembrane domain (Howard and Bechstedt 2004; Liang et al. 2013). The ankyrin domains in this repeat presumably form a superhelix as they arrange into a complete turn which can be easily deformed mechanically. Ankyrin domains are widespread across proteins with very different functions and occur in 0.85% of all metazoan proteins. However, the average number of ankyrin domains across all ankyrin-containing proteins is only 4.8 (±4.5, median 3), and proteins with more than ten ankyrin domains are rare (Jernigan and Bordenstein 2014) (see also fig. 1). Note that determining the precise number of domains in a domain repeat is a difficult problem. The underlying reasons are inaccuracies arising during sequencing and assembly, and rapid evolutionary changes of sequences and number of domains, even at the population level. Finally, computational challenges are common, for example, in the frame capturing of the domains in a repeat (Schaper et al. 2012). Consequently, even the most accurate programs may fail to determine the number of domains in a repeat by a number of 1. We will, in the following work, always use the lower number of prediction, for example, 28 if there might be 28 or 29 domains in a repeat.

TRP-N has been confirmed to be essential for many mechanosensory functions such as the control of body movement and the perception of touch in the fly (Effertz et al. 2011; Liang et al. 2013) and the nematode worm (Li et al. 2006), for maintaining equilibrium and hearing in zebrafish (Sidi et al. 2003), and it has also been localized in hair cells in the ear of frog (Shin et al. 2005).

So far, TRP-N has been reported neither in amniotic vertebrates nor in any nonbilaterian metazoan species (Venkatachalam and Montell 2007), meaning that its phylogenetic history and genetic origin remain unclear. As the understanding of the evolution of mechanoreception has important ramifications for understanding the evolution of organismic orientation and communication and thus the functioning of the neuronal system in general, it would be desirable to gain a broader understanding of TRP-N evolution, its phyletic distribution, and possible functional diversification. The recent publications of two cnidarian genomes, the fresh water polyp *Hydra magnipapillata* (Chapman et al. 2010) and the sea anemone *Nematostella vectensis* (Putnam et al. 2007), the placozoan *Trichoplax adhaerens* (Srivastava et al. 2008) and the sponge *Amphimedon queenslandica* (Srivastava et al. 2010) allow studying the deep phylogenetic roots of TRPs in general and, if existent in nonbilaterian Metazoa, of TRP-N in particular.

We here describe the evolutionary history of TRP-N across a representative set of metazoan genomes. We used custom-made Hidden Markov Models (HMMs), corrected several gene models of published genomes, resequenced Hydra-sequences, and thus revealed four previously unrecognized paralogs. We also inferred phylogenies of TRP-N and conducted sequence analyses to predict functionally significant sites. Furthermore, we present clues for the functional diversification of TRP-Ns in Hydra, based on their phyletic distribution, biophysical sequence analyses, and localization by in situ hybridization and immunohistochemistry. We propose a history of “rise and fall,” that is, the emergence, expansion and lineage specific loss of this protein family and a rationale of how a complex molecular trait related to mechanoreception became essential in some lineages but redundant in others.

## Materials and Methods

### Data Used

Genome and protein data for release 1.0 of the *Monosiga brevicolis* genome were obtained from the genome portal of the Joint Genome Institute (Nordberg et al. 2014; ftp://ftp.jgi-psf.org/pub/JGI_data/Monosiga_brevicollis/annotation/v1.0/). Release 2.2 of the *M. leidyi* genome was obtained from the download portal of the National Human Genome Research Institute (Ryan et al. 2013; http://research.nhgri.nih.gov/mnemiopsis/download/download.cgi?dl=genome, last accessed June 4, 2015). The 1.0 releases of the genomes of *H. magnipapillata*, *Branchiostoma floridae,* and *Saccoglossus kowalevskii* were obtained from the ftp of the National Center for Biotechnology Information (NCBI) (ftp://ftp.ncbi.nlm.nih.gov/genomes/). The genomes of *Capsaspora owczarzaki*, *Sphaeroforma arctica,* and *Salpingoeca rosetta* were obtained from the Broad institute (Origins of Multicellularity Sequencing Project, Broad Institute of Harvard and MIT, http://www.broadinstitute.org/). The *P. bachei* genome (Moroz et al. 2014) was obtained from http://rogaevlab.ru/pleurobrachia/data/genome.v1b.fa.gz, the *Acropora digitifera* genome (Shinzato et al. 2011) from http://marinegenomics.oist.jp/genomes/downloads?project_id=3, and the *O. carmela* genome from the Compagen (Hemmrich and Bosch 2008) website http://www.compagen.org/news.html. The *Schmidtea mediterranea* transcriptome (Abril et al. 2010) was obtained from https://planarian.bio.ub.edu/datasets/454/. Version 6.0 of the *X. laevis* genome was obtained from Xenbase (James-Zorn et al. 2013) (ftp://ftp.xenbase.org/pub/Genomics/JGI/Xenla6.0/). Genome and protein data for all other species were obtained from release 75 of the Ensembl database (Flicek et al. 2014; ftp://ftp.ensembl.org/pub/release-75/fasta/). (All web addresses were last accessed on June 4, 2015.)

TRP proteins are classified as follows:

1. TRP-N: The TRP-N ortholog in *D. melanogaster* was the first one that has been described, it was given the name *“*no mechanoreceptor potential C” (nompC, hence the “N” in TRP-N). This family is implicated in mechanosensation (Venkatachalam and Montell 2007).
2. TRP-C (canonical): Channels have diverse functions, but are generally activated by phospholipase C (Xu et al. 2008).
3. TRP-V (vanilloid): Activated through various mechanisms, many proteins of this subfamily are sensitive to temperature changes (Okazawa et al. 2002).
4. TRP-A: Named after the N-terminal ankyrin repeats (usually 11 ankyrin domains, average 8.2) and believed to be mechanical stress sensors (Nilius et al. 2007).
5. TRP-M (melastatin): Implicated in various biological functions ranging from cold sensation to regulation of cell adhesion, does not contain any N-terminal ankyrins, unlike most other TRP protein families. (Kraft and Harteneck 2005).
6. TRP-ML (mucolipin): Functionally not well characterized, mutations in human TRP-ML proteins are associated with lysosomal storage disorder mucolipidosis IV, a neurodegenerative disease (Nilius et al. 2007; Venkatachalam and Montell 2007).
7. TRP-PKD: Has been reported as the most ancient subfamily, with orthologs being identifiable in several microbial species such as *S. cerevisiae*. Mutations in human TRP-PKD proteins can cause polycistic kidney disease (Venkatachalam and Montell 2007).

A benchmark data set of TRP proteins was created by extracting proteins that have been annotated as belonging to TRP subfamilies from the Swiss-Prot database, using a custom Python script. A textfile with the benchmark data set has been uploaded with the supplementary material, Supplementary Material online, on bornberglab.org/links/trpn-evolution.

### Identification of TRP Family Members

Existing protein domain databases such as Pfam (Punta et al. 2012) do not provide HMMs which are specific for the TRP subfamilies (with the exception of the TRP-PKD subfamily, which is described by the Pfam model PF08016—“PKD_channel”). Pfam provides an unspecific HMM which matches not only many TRP channel domains from all subfamilies but also many non-TRP transmembrane domains (“Ion_trans”—PF00520). Therefore, we used the HMMER software package (Finn et al. 2011) to construct custom HMMs specific for each family. To accomplish this, we selected one protein sequence for each subfamily from the Swiss-Prot database which contains high-quality and experimentally supported gene model. We determined the transmembrane region of those sequences by predicting transmembrane helices with tmHMM (Krogh et al. 2001) and extracted the transmembrane region plus 20 amino acids adjacent toward the N- and the C-terminus. Specifically we selected the proteins O75762(TRP-A), P48995(TRP-C), Q7Z4N2(TRP-M), Q9GZU1(TRP-ML), Q9VMR4(TRP-N) and Q8NER1(TRP-V), the TRP-N protein is from *D. melanogaster*, all others are human proteins. These sequences were used as a query for a jackHMMER (Finn et al. 2011) search with five iterations and a stringent inclusion threshold of less than 1e-20 against the GenBank nonredundant protein data set (as of May 23, 2013; Benson et al. 2005).

For *Sc. mediterranea*, we could not identify a TRP-N locus in the published genome, but we could clearly identify the TRP-N domain in the TRP-N transcriptome (Abril et al. 2010) and thus conclude that *Sc. mediterranea* has TRP-N, but it is currently missing in the genome assembly.

All significant hits were combined, redundant hits removed, and the resulting set of sequences was aligned with MUSCLE (Edgar 2004). We used the SCI-PHY program for automatic subfamily detection (Brown et al. 2007) to predict subfamilies in this set of sequences. This yielded six large subfamilies (covering >90% of all sequences in the data set) and many very small subfamilies which likely correspond to spurious hits. We extracted the six largest clusters and used HMMbuild from the HMMER package (Finn et al. 2011) to create HMMs for each cluster.

We tested how well those six custom HMMs correspond to the six TRP subfamilies by scanning them against a benchmark set of all proteins that have been annotated as a member of one of the TRP subfamilies in Swiss-Prot. The HMMs discriminated between the members of the different subfamilies with 100% sensitivity and selectivity (specifically, each HMM yielded a significant *e* value [< 1e-10]) for all benchmark proteins of its respective subfamily. If more than one HMM yielded significant *e* values for a given sequence, it was always the family-specific HMM which yielded the most significant among these *e* values. Consequently, all custom HMMs produced neither false positives nor false negatives. Models are also provided online.

We then used our six custom HMMs together with the two previously mentioned PFAM HMMs to identify all TRP proteins in the NCBI nonredundant protein data set using hmmscan (*e*-value threshold < 1e-10) from the HMMER3 package (Finn et al. 2011). If the same protein sequence was identified as a significant hit by multiple HMMs, we assigned it to the subfamily that corresponds to the most significant hit. If the best hit corresponds to the previously mentioned unspecific HMM from Pfam (PF00520), we excluded the protein from further analysis because it is likely not a member of any TRP-family but rather belongs to a distantly related non-TRP ion channel family. For all significant hits, we extracted the region that aligns to the HMM (based on the “envelope” positions in the HMMER output), which corresponds to the channel region of the protein hits. In total, we identified 12,566 significant hits with an e-value threshold <1e-25 and at least 80% sequence coverage (i.e., putative hits are required to match at least 80% of the full-length domain sequence as defined by the HMM).

### Phylogenetic Tree Reconstruction of TRP Domains

We used USEARCH (Edgar 2010) to reduce the data set of 12,566 TRP channel regions to a smaller one which is suitable for phylogeny inference. Specifically, we collapsed the 12,566 hits into clusters of at least 80% intracluster pairwise sequence identity, which yielded 1,335 clusters. We also used USEARCH to extract the centroid sequence, that is, the representative sequence for the respective cluster (for a clustering at 80% identity, all centroid sequences are less than 80% identical to all other centroid sequences, and all sequences within a cluster are more than 80% identical to the respective centroid sequence of the cluster). We constructed a multiple sequence alignment of the centroid sequences with MUSCLE (Edgar 2004) and inferred a maximum-likelihood phylogeny based on this multiple sequence alignment with RAxML (Stamatakis 2014) running on the CIPRES cluster (Miller et al. 2011). We ran RAxML with the VT substitution model and the gamma model for rate heterogeneity, parameters which are most suitable for our alignment according to ProtTest (Darriba et al. 2011). We used the bootstopping feature of RAxML to let the program decide the required number of bootstrap replicates for obtaining stable support values. The phylogeny was visualized with SplitsTree (Huson and Bryant 2006) and is shown in figure 1.

### Correction of Erroneous Gene Models of TRP-N Homologs

TRP-N genes were not always correctly identified in the genomes which were used in this study (see above). In most cases, the start codons were not correctly identified and, accordingly, the resulting gene model missed several ankyrin domains. In some cases, sequences were not fully resolved in the assembly and filled up with placeholder “N”s. Therefore, the complete genomes were scanned with exonerate to identify open-reading frames with the potential to encode the TRP-N domain and, at the same time, showed signatures of at least some ankyrin domains. Exonerate (Slater and Birney 2005) was used with the parameters “exhaustive” (which turns all heuristics off and allows for the most sensitive search), “percent10” (which sets the required sequence identity threshold for putative hits to 10%), and “est2genome” (which models cDNA to genome alignments and allows for alignments to be interrupted by nonconserved regions such as introns). “MAKER” (Cantarel et al. 2008) was used to construct an improved gene model for the genomic region which contained the TRP-N locus by using the alignments of the TRP-N proteins of *Xenopus tropicalis, Danio rerio, D. melanogaster,* and *Caenorhabditis elegans*. A fasta file with the improved gene models is available in the supplementary material, Supplementary Material online.

### Phylogenetic Tree Reconstruction of TRP-N Proteins

We aligned a selection of the corrected TRP-N proteins with MUSCLE (Edgar 2004) and inferred a Bayesian phylogeny with the PhyloBayes program using the GTR (general time reversible) model (Lartillot et al. 2009). We used the automatic stopping rule feature of PhyloBayes and ran two chains in parallel until the maximum discrepancy between the columns of the trace files of the chains was less than 0.1 and the effective sizes of each column in the trace files were greater than 100. The phylogeny is shown in supplementary figure S6, Supplementary Material online; domain arrangements of the respective proteins were visualized with DoMosaicS (Moore et al. 2014) and projected on the phylogenetic tree.

### Identification of Mechanism-Specific Positions in the Alignment

**Fig. 2.—:**
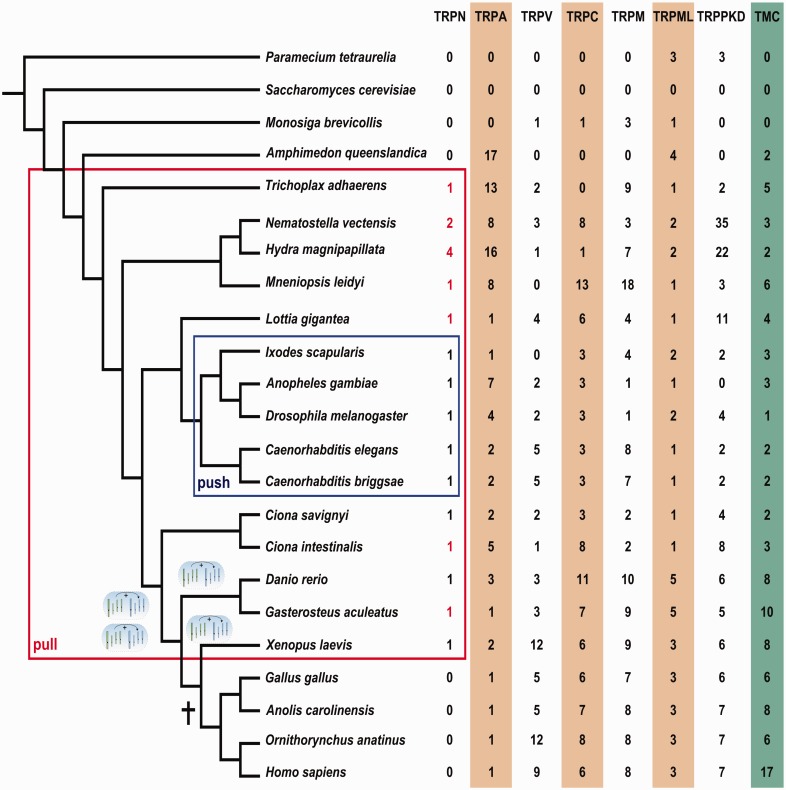
Phylogenetic distribution of transient receptor potential (TRP) families across Metazoa. The sizes of TRP subfamilies which were found using custom-made HMMs are listed at the tips of a phylogenetic tree for a representative set of metazoan genomes which were used (see Materials and Methods for a complete set of used genomes and supplementary fig. S4, Supplementary Material online, for corresponding phylogeny and occurrences of TRP-N). The tree topology is based on Philippe et al. (2011). Presumed events of WGDs are indicated by blue ellipses. Red frame encloses genomes in which TRP-N could be identified. Blue frame indicates TRP-N proteins which are activated through a “push,” mechanism (see text for explanations). Cross indicates the point at which the only bilaterian TRP-N copy has most likely been lost, that is, at the root of amniotes. TRP-N proteins with manually curated (in this study) gene models are in bold, and genes that were resequenced and PCR confirmed for this study are in red.

We used MUSCLE (Edgar 2004) to create a multiple sequence alignment of all TRP-N protein sequences mentioned in figure 2, except for the cnidarian ones for which it was a priori unknown whether they operate according to the push or pull mechanism. The resulting alignment was analyzed with JalView (Waterhouse et al. 2009; Troshin et al. 2011).

For all sequences except the cnidarian proteins, we calculated the conservation score per column in the alignment as implemented in JalView (based on Livingstone and Barton [1993]). This score takes biophysical similarities of amino acids into account and ranges from 0 for no conservation to 11 for perfect conservation.

We further applied the Multi-Harmony (Brandt et al. 2010) method to this alignment to predict the best candidates for residues that determine the functional specificity of the push and pull groups. This method calculates the “multi Relief” (mR) score for each site in the alignment. A high mR score indicates sites that are well conserved within groups but different between groups. The statistical significance of the mR score for each site is evaluated with a *z* score derived from a permutation test. We discarded all scores except those with a *z* score >3 and projected the remaining ones on the alignment in supplementary figure S3, Supplementary Material online.

We further filtered this set of statistically significant sites by only keeping those that are completely disjunct, that is, amino acids that occur in one group never occur in the other. For those sites, we determined whether the residues occurring in the cnidarian proteins fit the push group, the pull group, or neither one.

### Resequencing the TRP-N Loci in the Hydra Genome

Total RNA was isolated from whole animals of *H. magnipapillata* using the Trizol method (Life Technologies, Darmstadt, Germany). Oligo-dT primed first-strand cDNA synthesis was performed using SuperScript II reverse transcriptase (Life Technologies). Primers designed on *H. magnipapillata* sequence NW_002146487 (genebank), containing part of a TRP-N gene, were used to amp cDNAs by reverse transcription polymerase chain reaction (PCR). RACE (using 5′-RACE System; Life Technologies) was used to amplify 5′-exons. PCR fragments were PCR-amplified using GoTaq enzyme (Promega, Madison, WI), cloned with the pGEM-T vector system (Promega), and sequenced. Sequences obtained were used to identify additional genomic sequences coding for TRP-N genes. Assembly of full length open-reading frames for three additional genes was done accordingly. Results are provided online.

### Antibody Staining and In Situ Hybridization of TRP-N Paralogs in Hydra

#### In Situ Hybridization

Locked nucleic acid (LNA) probes with double-DIG (dioxygenin) labeling (5′-DIG and 3′-DIG) were created by Exiqon based on DNA sequences of TRP-N1 and TRP-N2. The LNA probes were diluted with hybridizing solution to approximately 0.77 and 2.55 µM for TRP-N1 and TRP-N2, respectively, and then used in a 1:500 dilution for the experiment. Animals were relaxed with 2% urethane in Hydra medium and fixed overnight with freshly prepared 4% paraformaldehyde (PFA) in Hydra medium. The fixed animals were transferred to 100% ethanol and rehydrated in 5-min steps using 75%, 50%, 25% ethanol in PBS, 0.1% Tween20 (PBST, phosphate buffered saline with Tween 20). After three 5-min washing steps with PBST the animals were incubated with 1× Proteinase K in PBST for 7 min. The reaction was stopped by adding 4 mg/ml glycine in PBST. Then, the animals were equilibrated in 0.1 M triethanolamin (TEA) for 2 × 5 min and incubated for 5 min each with 0.25% and 0.5% acetanhydride in TEA, followed by two washing steps with PBST. Then, a refixation with 4% PFA was performed for 20 min at room temperature, followed by five 5-min washing steps with PBST. The animals were incubated with hybridizing solution (50% formamide, 5 × SSC [0.75 M NaCl, 0.075 M trisodium citrate, pH 7.0], 1 × Denhardt’s [1% polyvinylpyrrolidone, 1% ficoll, 1% BSA], 200 µg/ml yeast RNA, 100 µg/ml heparin, 0.1% Tween20, 0.1% CHAPS [3-[(3-cholamidopropyl)dimethylammonio]-1-propanesulfonate], 10% H_2_O) for 10 min and then prehybridized in hybridizing solution for 2 h at 55 °C. The probes were diluted in hybridizing solution and denatured by heating (70 °C, 10 min). The animals were incubated with the probes for 2.5 days at 50 °C for TRPN1 and 55 °C for TRPN2. Unbound probes were removed by 5-min washing steps with 100%, 75%, 50%, 25% hybridizing solution in 2 × SSC followed by two incubations for 30 min in 2 × SSC, 0.1% CHAPS. The animals were equilibrated in maleic acid buffer pH 7.5 (MAB: 100 mM maleic acid, 150 mM NaCl) for 2 × 10 min and blocked in 1% blocking reagent (Roche) in MAB for 2 h at room temperature. An anti-DIG antibody coupled to alkaline phosphatase was used at 1:4,000 in blocking solution at 4 °C overnight. Unbound antibody was washed out during eight 30- to 60-min washing steps with MAB, followed by an overnight washing step. To detect the signal, the animals were first equilibrated 2 × 10 min in NTMT (100 mM NaCl, 100 mM Tris pH 9.5, 50 mM MgCl2, 0.1% Tween20) at room temperature and then incubated in NBT/BCIP (nitro-blue tetrazolium/5-bromo-4-chloro-3'-indolyphosphate) (Roche, premixed solution) 1:50 in NTMT in the dark at 37 °C. In some cases, separate NBT and BCIP solutions (Roche) were used. 3.75 µl of each stock solution was added per ml staining solution. When reaching the optimal signal to background ratio, the reaction was stopped by adding 100% ethanol. The animals were rehydrated by incubation for 5 min in 75%, 50%, and 25% ethanol in 0.1 × PBS. After a final rehydration step in PBS the animals were mounted on microscopic slides in PBS 90% glycerol.

#### Immunocytochemistry

*Hydra magnipapillata* were relaxed in 2% urethane in Hydra medium and then fixed in freshly prepared ice-cold methanol for 4 h (pan-TRP-N) or overnight (TRP-N4) at 4 °C. Samples were rehydrated in 5-min steps using 75%, 50%, 25% ethanol in PBS, washed three times in PBS, then incubated in PBS 0.1% Triton X100 for 30 min. Samples were incubated in PBS with 1% BSA for 1 h before being incubated overnight at 4 °C in the same solution with the antibody. To remove unbound antibodies, three 30-min washing steps with PBS were performed. The incubation with the secondary antibodies was performed for 2 h at room temperature. The secondary antibodies were diluted 1:400 in PBS 1% BSA. To remove unbound antibodies, the animals were washed three times with PBS and then mounted on object slides with PBS 90% glycerol.

#### Decnidocilation

Cnidocils were removed following the procedure of Golz and Thurm (1990). Isolated cnidocils were fixed in a final concentration of 1% PFA for 15 min before being transferred to poly-l-lysine (Sigma-Aldrich) coated slides. The solution was allowed to settle for 1 h before being washed in PBS. Slides were incubated in PBS with 1% BSA for 30 min before being incubated with antibody in the same solution for 30 min. Slides were washed in PBS and then incubated with secondary antibody (Alexa fluor goat antirabbit 488) diluted 1:500 in PBS with 1% BSA for 30 min. After washing with PBS, slides were allowed to partially dry before cover slips were mounted. All incubation steps occurred at 37 °C.

#### Scanning Electron Microscope

Live specimens of *Hydra vulgaris* were fixed in 2.5% glutaraldehyde in phosphate buffer at pH 7.4 and postfixed in 1% OsO4 in phosphate buffer. The specimens were dehydrated in a graded ethanol series, critical point dried with CO_2_, then sputter-coated with gold palladium with a Hummer sputter coater and examined using a LEO 1550 field emission scanning electron microscope at the University of Kansas, Lawrence.

#### Phalloidin Staining of Stereocilia

*Hydra magnipapillata* were relaxed in 2% urethane in Hydra medium and then fixed in freshly prepared ice-cold 4% PFA overnight at 4 °C. Samples were prepared as for single stainings as detailed above. However, in addition to the secondary antibody for pan-TRP-N, the samples were also incubated with Alexa Fluor 488 Phalloidin (1:500) in PBS, 1% BSA. Final washing and mounting steps remained the same as for single stainings.

## Results and Discussion

### Evolution of TRP Subfamilies

To reconstruct the evolutionary history of TRP-N, we first searched for homologs of all TRP subfamilies across Metazoa. We analyzed genomes from a representative set of Metazoa which comprised Placozoa, Cnidaria, Ecdysozoa (molting Metazoa with a three-layer cuticula, including Arthropoda), Lophotrochozoa, and Chordates. As an outgroup we used *Saccharomyces cerevisiae*. A full account of used genomes is given in Materials and Methods.

The Pfam database (Punta et al. 2012) provides one HMM that specifically matches the channel regions of the TRP-PKD (polycystic kidney disease) subfamily and one unspecific HMM which is designed to find various ion channels and matches most sequences from the TRP family. Therefore, we built custom HMMs for all TRP subfamilies other than TRP-PKD. Starting with the transmembrane regions from one member of each TRP subfamily from Swiss-Prot, we performed an iterative jackHMMER scan (Finn et al. 2011). TRP sequences, including 25 amino acid windows flanking the TRP domain region C- and N-terminally, were aligned using MUSCLE (Edgar 2004) and subfamilies were predicted with SCI-PHY (Brown et al. 2007). We manually constructed new HMMs based on the six largest subfamilies and tested their specificity by scanning a benchmark set of all proteins that have been annotated as a member of one of the TRP subfamilies in Swiss-Prot (see Materials and Methods for details). The HMMs discriminated between the members of the different subfamilies with 100% sensitivity and selectivity (i.e., no false positives or negatives). We used hmmscan (Finn et al. 2011) to scan with our six custom HMMs and the previously mentioned two HMMs provided by Pfam against the GenBank nonredundant protein data set. This scan produced 12,566 significant hits. For each hit, we extracted the region that matched the HMM for further analysis. To reduce this data set to a smaller size suitable for phylogeny inference, we used USEARCH (Edgar 2010) and collapsed the 12,566 hits into clusters of at least 80% intracluster pairwise sequence identity, resulting in 1,335 clusters. For each cluster, we extracted the most representative sequence (see Materials and Methods), aligned them with MUSCLE (Edgar 2004) and used the resulting multiple sequence alignment to infer a maximum-likelihood tree with RAxML (Stamatakis 2014). All members within a subfamily grouped together (see fig. 1), which adds further confidence to the high quality and discriminative potential of the HMMs which were used.

All TRP subfamilies except for TRP-A and TRP-N can be identified in *S. cerevisiae* or *Paramecium tetraurelia* and thus appear to be ancient. TRP-N, however, has presumably emerged only after the splits between Porifera, Eumetazoa (Bilateria, Cnidaria), and Placozoa (see below for further phylogenetic considerations and implications).

### Conservation and Variation of Domain Arrangements in TRP Subfamilies

We next analyzed the domain arrangements of all TRP subfamilies to determine how conserved these arrangements are within and between the respective subfamilies. Domains are evolutionary units of proteins (Moore et al. 2008, 2013; Forslund and Sonnhammer 2012) and their presence/absence patterns are strong phylogenetic markers albeit at a much longer time scale than absence/presence and insertion/deletion patterns at the amino acid level (Yang et al. 2005).

We find two remarkable properties: First, the intrafamily divergence in terms of domain-arrangement similarities is very low for TRP-N, much lower than for all other TRP subfamilies. Second, in all TRP-Ns for which full-length gene sequences were present or where the gene models could be manually reconstructed (see Materials and Methods) the number of ankyrin domains is invariably approximately 28 (see above for difficulties arising in determining the precise number of domain repeats). We do find TRP-N proteins with fewer than 27 ankyrin domains (see fig. 1); however, all of these cases correspond to fragmented gene models that lack one or more exons and have no or incorrectly annotated start codons. It is intriguing that the TRP-N subfamily shows such a high degree of conservation in terms of the ankyrin repeat because repeat containing proteins in general and ankyrin domain containing repeats in particular have a high tendency to rapidly change the number of repeat domains, even over time scales which are relatively short compared with those which apply to other domain rearrangements (Verstrepen et al. 2005; Björklund et al. 2006; Gemayel et al. 2010; Jernigan and Bordenstein 2014; Schaper et al. 2014). The degree of conservation in terms of the number of ankyrin domains over the long evolutionary timespan of TRP-N (roughly 715 Myr, i.e., since the presumed split between Placozoa and all other Metazoa [Erwin et al. 2011]) and against the backdrop of highly divergent intron–exon boundaries (see supplementary fig. S2, Supplementary Material online) in TRP-N is thus remarkable. Accordingly, TRP-N is presumably under much stronger selection to maintain its long repeat of ankyrin domains than many other repeat containing proteins and in particular other ankyrin containing proteins (see also bar plot labeled “all” in fig. 1), including the other TRP subfamilies (other bar plots). The most likely reason would be that TRP-N has to conserve its structure in order to maintain its function which is essential for survival or otherwise would be rapidly weeded out. This explanation complies with the proposed and aforementioned structure of a superhelix, because approximately 28 ankyrin domains are required for one full helical turn which in turn allows transmission of force from one end of the structure to the other without creating a “torque” (Howard and Bechstedt 2004). It is thus unlikely that TRP-N can tolerate large deviations in the number of ankyrin domains without losing functionality of the gating spring.

Second, the number of approximately 28 ankyrin domains in TRP-N is the highest among all TRP proteins (see fig. 1). As gain and loss of domains are very rare events (Moore and Bornberg-Bauer 2012), the most parsimonious evolutionary scenario is that ankyrin repeats have been gained by an ancestral transmembrane protein after its split from the ancestors of the TRP-ML subfamily and were retained in all subfamilies except the TRP-M subfamily. In TRP-N, the ankyrin repeats probably expanded and, after the superhelical structure was established and became beneficial, the number of ankyrin domains became fixed.

High conservation in TRP-N is also observed at the sequence level, especially in the C-terminal domain (see supplementary fig. S3, Supplementary Material online) which contains several motifs that are identical between all studied orthologs. An example is the long TRP motif VLINLLIAMMSDTYQRIQ, very close to the C-terminus.

### Loss, Retention, and Expansion of TRP-N

Another indicator of a protein’s functional relevance for organisms along a lineage is the expansion of its protein family by gene duplication or, to be more precise, the retention of duplicates over evolutionary long time scales. Conversely, if copies after single gene duplications or whole-genome duplication (WGD) are frequently lost, this may also indicate that even small mutations will easily render a protein nonfunctional. Consequently, such a nonfunctional copy will be rapidly weeded out. In the evolutionary history of the TRP-N subfamily, we find both such signals.

We searched with the custom-made HMMs against the official gene sets of the genomes in our data set. To catch putative TRP-N homologs which may have been missed in the gene prediction pipelines, we also scanned the raw genome assemblies for putative TRP-N loci with exonerate (Slater and Birney 2005) and constructed custom TRP-N gene models for species that had a clearly identifiable TRP-N homolog, but no corresponding gene model in the official gene set. For most genomes, we found exactly one TRP-N candidate with perfectly or close to perfectly conserved domain arrangement. All TRP-N homologs with fewer than 27 ankyrin domains corresponded to fragmented gene models that lacked one or more exons, had falsely predicted start codons or several ORFs were collapsed into one. For those cases, we tried to correct the gene models using exonerate and Maker (Cantarel et al. 2008).

In Cnidaria, we observed two remarkable differences: There were four spurious hits to the TRP-N transmembrane domain in *H. magnipapillata* and two such hits in *N. vectensis*. In all these proteins, there were much fewer than 27 ankyrin domains in the repeat and we were unable to correct the gene models with bioinformatics methods because the respective loci were not fully resolved in the published genome assemblies and were filled with long stretches of placeholder Ns. Consequently, we designed primers and sequenced all four Hydra genes in their entire length (with the exception of one exon in one gene, see Materials and Methods for details), thus bridging many long introns (see supplementary fig. S2, Supplementary Material online). All full length hydra TRP-N genes encode for approximately 28 ankyrin domains. Reconstruction of full length gene models was not possible for all genomes. The TRP-N loci in the two ctenophora genomes used in this study were also not fully resolved in the published genome assemblies and would have to be resequenced before building complete TRP-N gene models.

The sizes of all TRP protein families, that is, the number of family members which can be found in one genome vary strongly (see fig. 2). For example, there are between zero and up to 35 TRP-PKD copies. Considering the limited quality of several genomes and the relatively small family sizes, any sign of absences in single genomes must be interpreted with caution (Clamp et al. 2007; Milinkovitch et al. 2010; Elsik et al. 2014). However, as these variations are consistent across several subfamilies and genomes, and as our custom-made HMMs have a high discriminative power (see above), it is unlikely that artifacts contribute significantly to these variations.

Unlike other TRP subfamilies, TRP-N occurs with exactly one copy in all Bilateria except for Amniota. This consistent occurrence of a single-copy TRP-N is in contrast to all other TRP subfamilies. As gene duplication per se is a predominantly stochastic process (Blomme et al. 2006), the dynamics of loss of duplicates in TRP-N seems to be different from other TRP proteins. Identical copies of genes tend to be lost (Chain et al. 2011) unless they acquire a new beneficial mutation (sub- or neofunctionalization) (Chain and Evans 2006), whereas copies with detrimental mutations will be even more rapidly lost and not be fixed. Apparently, adaptive mutations are less likely to occur in TRP-N and these stronger functional constraints can be rationalized by the afore mentioned structural requirements to form a superhelix which is unique for TRP-N among the TRP subfamilies.

In particular, a reset to a single copy can also be observed after all events of WGDs (see fig. 2) which occurred along the evolutionary history of vertebrates, that is, after the two WGDs at the root of vertebrates, in the bony fish lineage and even after the very recent WGD in the lineage of the clawed frog *Xenopus laevis*. The other TRP families show much more variation of family size, which is the norm for protein families in the aftermath of a WGD (Maere et al. 2005; Chain et al. 2011).

### Ramifications of TRP-N Copy Numbers for Deep Metazoan Phylogenies

The occurrence of one TRP-N copy in the placozoan *T. adhaerens* and the ctenophores *Pleurobrachia bachei* and *Mnemiopsis leidyi*, coupled with the absence in the sponge *A. queenslandica* (see fig. 2 and supplementary fig. S4, Supplementary Material online), is particularly intriguing for a couple of reasons. First, it is remarkable to find such a highly sophisticated membrane protein which requires an elaborate complex of other proteins for function (see supplementary fig. S1, Supplementary Material online) in an organism which is deeply rooted in the metazoan lineage and which is supposed to have no more than three cell types. One of these cell types is supplied with a motile cilium (Ruthmann et al. 1986).

Second, the correct phylogenetic relationships between sponges, Cnidaria, Bilateria, and Placozoa are still debated (Collins 2002; Dunn et al. 2008; Schierwater et al. 2009; Philippe et al. 2011; Nosenko et al. 2013; Ryan et al. 2013; Moroz et al. 2014) (see also supplementary fig. S4, Supplementary Material online). In particular, the recent publication of two ctenophore genomes (Ryan et al. 2013; Moroz et al. 2014) suggested that Ctenophora may be an outgroup to all other Metazoa. Such a grouping would suggest that Ctenophora have developed mesoderm-like features and a simple neuronal system, independently from other Metazoa. While bearing in mind that the presence/absence pattern of a single gene is of course error prone and gives only weak statistical support on its own, the absence of TRP-N in the sponges *A. queenslandica* and *Oscarella carmela* provides further, albeit limited, support for sponges being the sister group of all other Metazoa (Philippe et al. 2011). Note that this interpretation is independent of whether sponges form a monophyletic or paraphyletic group (Collins 2002; Jackson et al. 2007; Sperling et al. 2009; Erwin et al. 2011) but places Placozoa as the outmost group within all other Metazoa, and sponges as an outgroup to this common clade (see fig. 2).

### Diversification between the TRP-N Paralogs in Hydra

We further investigated the possibly diverged functions of the four TRP-N paralogs in Hydra. Typically, an exceptional retention of paralogs is a consequence of a newly gained function which may confer additional fitness and prevents the underlying gene from being weeded out again (Dittmar and Liberles 2010; Chain et al. 2011; Sikosek et al. 2012). The underlying mechanistic processes may be manifold and include, among others, neofunctionalization (Boucher et al. 2014) and subfunctionalization (Chain and Evans 2006), for example, due to newly attained biochemical functions (Ganfornina and Sánchez 1999), or new genomic (Abascal et al. 2013) or cellular (Soskine and Tawfik 2010) environments.

One change of functionality within the TRP-N family was the shift from the pull to a push mechanism (see Introduction). We compared the protein sequences within and between the push and pull group of TRP-Ns and identified residues which are consistently similar within but different between these two groups. We found 17 residues to be highly discriminative between the proteins from the push and the pull group (see supplementary fig. S3, Supplementary Material online). In all four Hydra paralogs (hydra-TRP-N1–4, see Materials and Methods for details), all of these residues comply with a pull mechanism.

**Fig. 3.—:**
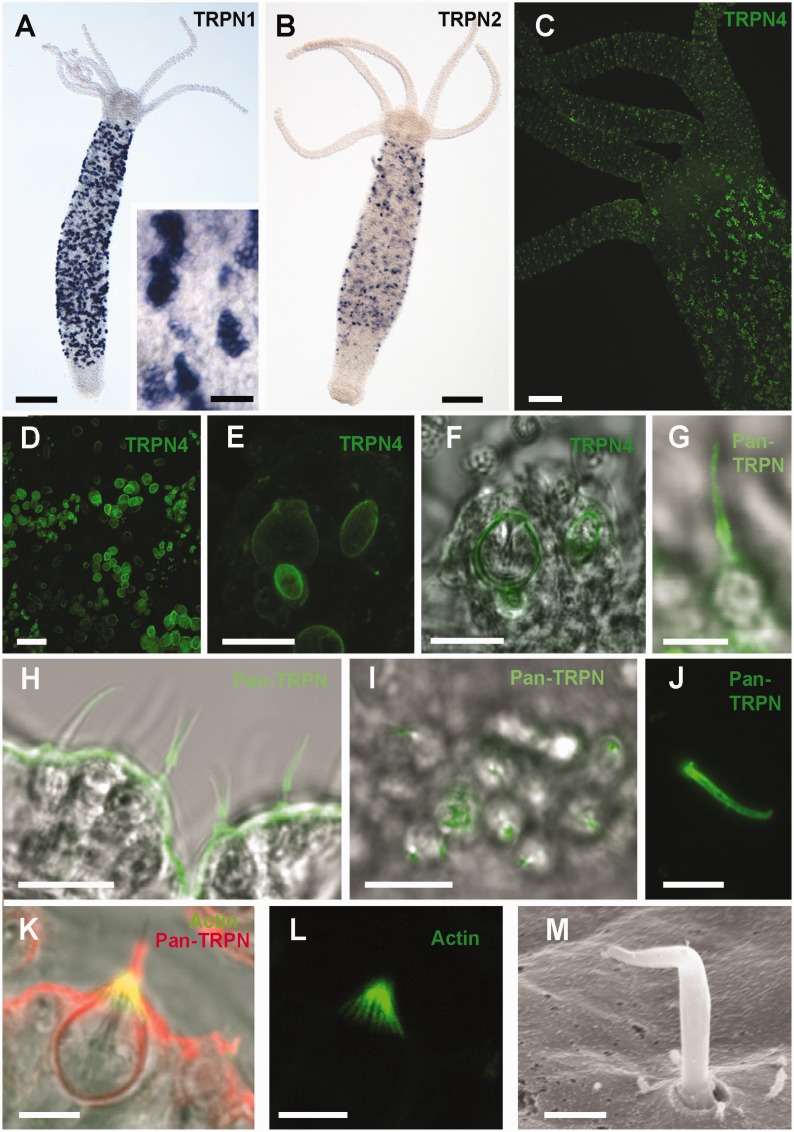
Expression and localization of individual TRP-N molecules in *Hydra magnipapillata.* (*A–F*) In situ hybridizations. (A) In situ hybridization for TRP-N1 showing expression of transcripts in the nests of developing nematocytes. Scale bar = 100 µm. Inset shows a close up of TRP-N1 positive individual nematocyte nests in the body column of the same animal as in (*A*). Scale bar = 50 µm. (*B*) In situ hybridization for TRP-N2 showing expression of transcripts in developing nematocytes, Scale bar = 100 µm. (*C–F*) Immunostaining with TRP-N4 antibodies. (*C*) Overview of localization of TRP-N4 protein in tentacles and body column by antibody staining. Scale bar = 100 µm. (*D*) Localization of TRP-N4 in the nests of developing nematocysts in the body column. Scale bar = 20 µm. (*E*) Localization of TRP-N4 in tentacles where it surrounds individual mature nematocyst capsules. Scale bar = 10 µm. (*F*) Transmitted light view on nematocysts in (*E*) showing localization around various nematocysts. Scale bar = 10 µm. (*G–M*) Immunostaining of cnidocils with pan-TRP-N antibody. (*G*) A single nematocyte with desmoneme and stained cnidocil. Scale bar = 5 µm. (*H*) Staining of cnidocils in tentacle. Scale Bar = 10 µm. (*I*) Surface view of tentacle indicating spot-like staining in the basal part of cnidocils. Scale Bar = 10 µm. (*J*) Staining of isolated cnidocil showing a gradient toward the base of the cilium. Scale bar = 10 µm. (*K*) Costaining of TRP-N in the cnidocil (red) and of phalloidin in the stereocilia (green). Scale bar = 5 µm. (*L*) Same nematocyte as in (*E*), showing phalloidin staining of the stereocilia only. Scale bar = 5 µm. (*M*) Scanning electron microscope of tentacle surface with cnidocil. Scale bar = 2 µm.

We next aimed to localize expression of TRP-Ns and distinguish possible spatial differences in their expression. In situ hybridization (see Materials and Methods for details) was used to localize TRP-N1 and TRP-N2 mRNAs. Due to a lack of unique sequences, specific probes for the other TRP-N mRNAs were not feasible. Expression patterns of both hydra-TRP-N1 and hydra-TRP-N2 reveal a clear restriction to developing nematocytes in the body column of hydra, with hydra-TRP-N1 showing a stronger signal than Hydra-TRP-N2 (see fig. 3*A* and *B*). This strong hydra-TRP-N1 signal (see fig. 3*A*) refers to cell clusters of nascent, premature nematocytes (Fawcett et al. 1959). These clusters are known to break up upon maturation and the isolated nematocytes subsequently migrate toward the tentacles (Campbell and Marcum 1980). This expression pattern of hydra-TRP-N1 and hydra-TRP-N2 resembles the ones of most nematocyst-associated genes, such as minicollagens, which are downregulated in the head region (Beckmann and Özbek 2012).

For antibody design, we scanned the four hydra-TRP-Ns for sequence fragments that are highly specific and cannot be found in any other Hydra protein. As this was only successful for hydra-TRP-N4 and N1, a second, “generic” antibody was raised (pan-TRP-N) against a consensus sequence, which is present in a region overlapping one of the ankyrin repeats in all four hydra-TRP-Ns (see supplementary fig. S5, Supplementary Material online). Staining for hydra-TRP-N4 showed localization to nematocytes, both in clusters of nascent nematocysts and in tentacles (see fig. 3*C*). The signal was restricted to the vesicle membrane surrounding the nematocyst capsule (see fig. 3*D*–*F*). Interestingly, mostly clusters with mature nematocysts containing large capsule vesicles, and mature nematocysts in the tentacles were detected (see fig. 3*D*–*F*). Immunostainings performed with the generic pan-TRP-N antibody showed a distinct pattern from the hydra-TRP-N4 stainings because nematocyst capsules were only weakly stained. The dominant signal for the pan-TRP-N antibody was located at the mechanosensory cnidocil apparatus, in the tentacle (see fig. 3*G* and *H*). The staining was restricted to the central cilium with a stronger intensity toward its base (see fig. 3*G*–*J* and supplementary fig. S1, Supplementary Material online). This gradient was further confirmed with enhanced stainings of isolated cnidocils (see fig. 3*J*). A costaining with phalloidin revealed a clear distinction from the stereovilli surrounding the central cilium (see fig. 3*K*–*M* and schema in supplementary fig. S1, Supplementary Material online). Taken together, the results from the identification of group-specific residues, immunostainings, and in situ hybridizations suggest a strong functional diversification between the hydra-TRP-N paralogs which can, however, only be understood by delineating the detailed ultrastructure which underlies the function of mechanosensitive channels (Golz and Thurm 1991b; Brinkmann et al. 1996; Thurm et al. 2004). We find that the pan-TRP-N antibody signal is localized to the mechanosensory cnidocil apparatus of all types of nematocytes in the tentacles, whereas hydra-TRP-N4 is restricted to nematocyst vesicle membranes in developing nematocyte clusters in the body column of Hydra and in mature nematocytes of tentacles (see fig. 3*C*–*F*). Nematocysts are enclosed by a plasma membrane, which ensheathes a massive collagenous wall (Özbek 2011). This wall sustains an extreme internal pressure of 150 bar in stenotele nematocysts (Weber 1989), which discharge upon touch and are essential for predation and defense in Hydrozoa.

Outside, the cyst membrane is enclosed in a basket of microtubules to which the membrane is densely connected by periodic bridges (Golz 1994). In length, diameter, and periodicity, these bridges resemble the membrane–microtubule connectors of the mechanosensory membrane of insect sensilla (Thurm et al. 1983), which have been identified to be TRP-N (Liang et al. 2013) (for a schematic drawing, see supplementary fig. S1, Supplementary Material online). Therefore, the binding of TRP-N4-antibody likely detects TRP-Ns in the basket connectors of the cnidocyst membrane. This suggests that the cnidocyst membrane is studded with mechanosensitive cation channels at a density of approximately 1,600/µm^2^ (Golz 1994). These cation channels open their conductance in response to cyst-diameter changes in the nanometer range. The comparison of the TRP-N4-sequence with the push/pull-correlated differences in noncnidarian sequences (see supplementary fig. S3, Supplementary Material online) may indicate opening by a pulling force, that is, reduction of cyst diameter. The TRP-N type of channel suggests a particularly short latency of opening, as is also known from sensory responses of insects (≤20μs, see Thurm et al. 1983) and hydrozoans (≤50μs, see Brinkmann et al. 1996). This fast response is important to compete with the high-speed kinetics of volume changes during capsule discharge (Holstein and Tardent 1984). Thus, not all TRP-N in Cnidaria are sensing external forces. Instead, the extraordinary arrangement of TRP-N4 around the capsules suggests that TRP-N4 is engaged in coupling the permeabilities of ions and accompanying water in the membrane barrier to the changes in diameter and pressure of the capsule in the processes of charge or discharge (i.e., electro-osmotic ion-water coupling, see, e.g., Küppers et al. 1986). Although leading components of these processes have been discovered, other essential parameters are still unknown, precluding a modeling of the processes in full detail (reviewed in Berking and Herrmann 2005; Tardent 1995). The association of a TRP-N paralog with stenoteles, the Hydrozoa-specific nematocysts, therefore rationalizes the benefits of at least one duplication of the TRP-N gene as a feature of cnidarians, which is unique in the animal kingdom.

## Conclusions

Mechanosensation is among the foremost means of communication between cells and with the environment and therefore of paramount importance for the evolution of multicellularity and a complex bauplan. Although it does not operate through a second messenger system like visual and olfactory receptors, mechanosensation in most cases uses a sophisticated apparatus of many intricately arranged proteins acting in highly concerted manner (see supplementary fig. S1, Supplementary Material online). One protein will lack functionality without any of the others it interacts with and our studies demonstrate that such a complex apparatus involving TRP-N must have been present at or near the root of all multicellular Metazoa. The importance of TRP-N is further supported by its strong conservation in domain arrangement and copy number across all lineages. Both observations reveal a contrast to the other TRP ion channel proteins.

The expansion of TRP-N in hydra and, to a lesser extent in Nematostella, is a striking exception. It bears strong evidence of functional paralogs which have conferred additional functionality. The diversification of hydra-TRP-N4 (resp. its ancestor) may have been essential for the success of developing the cnidocyst weaponry and thus the success of this cnidarian-specific route of evolution. Similar conclusions may hold for the anemone *N. vectensis* although the two TRP-N paralogs in its genome emerged from a gene duplication event that occurred independently after the split of the *N. vectensis* and *H. magnipapillata* lineages (see supplementary fig. S6, Supplementary Material online). The second duplication in Hydra and a closer relationship between hydra-TRP-N1 and hydra-TRP-N3 corresponds with a particularly evolved sensing apparatus in Hydrozoa (Golz and Thurm 1991a; Holtmann and Thurm 2001).

The complete loss of TRP-N at the root of Amniota has been preceded by an increase in numbers of stereovilli per cell, which occurred with a change from the originally concentric organization of hair bundles to their eccentric organization at the origin of vertebrates (Hudspeth and Jacobs 1979). This could occur as stereovilli themselves are potential contributors of mechanosensation, observed already in the Cnidaria *Nematostella* (Mire and Watson 1997) and *Hydra* (Thurm et al. 2004). Stereovilli, however, like all microvilli, are supported by an actin skeleton instead of the tubulin skeleton of cilia that supports TRP-N (see supplementary fig. S1, Supplementary Material online, and fig. 3*K* and *L*). The mechanotransducing channels best favored for vertebrate stereovilli at present are TMC channels (Morgan and Barr-Gillespie 2013; Pan et al. 2013), possibly associated with actin. TMCs have presumably arisen at the root of metazoan too but expanded significantly in vertebrates, probably in the wake of the two rounds of WGDs (see right column in fig. 2). In the transduction of vertebrate hair cells, TMCs apparently took over the dominant role for mechanosensation. Indeed in frog, though TRP-N is still present, a functional contribution of TRP-N has not been found in saccular hair cells (Shin et al. 2005) while TMC containing stereovilli in hair cells respond to mechanical stimulation (Hudspeth and Jacobs 1979). One can thus speculate that the stage for the loss of TRP-N in Amniota has been set by the expansion and/or specialization of TMC proteins.

In summary, the rise and fall of TRP type mechanoreceptors tell an intriguing story of how complex traits evolve, diversify, and become redundant. Several of the results presented here, such as the key residues which distinguish the push and pull mechanisms, should pave the way for and help direct further computational and experimental research toward a better understanding of the detailed molecular properties underlying mechanosensation.

## Supplementary Material

Supplementary figures S1–S6 are available at *Genome Biology and Evolution* online (http://www.gbe.oxfordjournals.org/).
